# The National Egg Collection for the Wounded

**Published:** 1916-06-17

**Authors:** R. J. Dartnall

**Affiliations:** Hon. Secretary. 154 Fleet Street, E.C.


*To the Editor of* The Hospital.

### The National Egg Collection for the Wounded.

*To the Editor of* The Hospital.

Sir,—The attention of the executive committee of the National Egg Collection has been drawn to a reference to their work appearing in your issue dated May 27.

I have been instructed to write and inform you that the paragraph is a gross misrepresentation of the facts, and I am to request you to be good enough to do them the justice of immediately contradicting the statement, which is untrue, that the Army Council has taken steps to secure publication of the accounts. The inference which the writer of the paragraph seeks to draw, that the committee of the National Egg Collection were averse to the publication of the accounts, but that owing to the action of the Army Council the accounts are now to be published, is absolutely unfounded.

The committee have always been desirous of publishing the accounts, and it is solely due to their representations to the Army Council, and not to the action of Sir Norval Helme or any other person, that the accounts are now under consideration by an independent firm of chartered accountants, with a view to their early publication.—Yours faithfully,

The National Egg Collection for the Wounded.
R. J. Dartnall, Hon. Secretary.
154 Fleet Street, E.C., June 5, 1916.

[The above letter, though dated June 5, did not reach the offices of The Hospital until June 8, and was therefore too late for inclusion in our last issue. We are glad to have the committee's assurance that they have always been desirous of publishing the accounts. We hope that there will now be no delay in their early issue to the public.—Ed. The Hospital.]

